# Municipal Housekeeping in Paris

**Published:** 1896-02

**Authors:** 


### Municipal Housekeeping in Paris.

Paris has spent an immense sum in admirable public markets, but she now receives as rental from them annual income of more than 8,000,000 francs. The huge La Villette abattoir has not only been advantageous from a sanitary point of view, but it yields the city an income of about 3,000,000 francs a year. Facts like these show that Paris knows how to make her municipal housekeeping pay as well as yield large and splendid public results.—*British Medical Journal.*

Children's Food.—Dr. Allen does not approve of giving little children coarse, hard food to masticate. The temporary teeth are not suited to that purpose from their brief duration, during the early part of which the roots are undeveloped, and the teeth not firmly fixed in the alveolus, while during the latter portion of of their retention the roots are being absorbed, the edges having sharp, jagged points not fitted to bear the pressure of excessive mastication. The effort being painful, the child swallows its food as best it can, and the foundation for dyspepsia is early laid.

